# HK3 is correlated with immune infiltrates and predicts response to immunotherapy in non‐small cell lung cancer

**DOI:** 10.1002/ctm2.6

**Published:** 2020-05-07

**Authors:** Zhan Tuo, Xin Zheng, Yan Zong, Jie Li, Chunyan Zou, Yi Lv, Jun Liu

**Affiliations:** ^1^ Cancer Center Union Hospital Tongji Medical College Huazhong University of Science and Technology Wuhan China; ^2^ Department of Pharmacy, Union Hospital, Tongji Medical College Huazhong University of Science and Technology Wuhan 430022 China; ^3^ The People's Hospital of Guangxi Zhuang Autonomous Region Nanning Guangxi 530021 China

**Keywords:** HK3, immune infiltrates, immunotherapy, NSCLC

## Abstract

**Background:**

With the knowledge of tumor immunobiology deepening among researchers, the breakthroughs in the field of tumor immunotherapy in recent years have provided new approaches for cancer therapy. While patients who receive treatment are all at risk of side effects, about one‐fifth of them have sustained responses. It is crucial to figure out the underlying mechanism of how the immune system regulates the nonsmall cell lung cancer (NSCLC) microenvironment to improve the benefit of immunotherapy. Regarding glucose metabolism, the initial step is to generate glucose‐6‐phosphate by phosphorylating glucose with hexokinases‐3 (HK3). According to a recent study, HK3 has a functional role in the treatment of acute promyelocytic leukemia and colorectal cancer.

**Results:**

Here, we studied the co‐expression relationship between the glycolytic pathway gene and the immune checkpoint gene and found that the expression of HK3 in tumor tissues may be related to immune status. By analyzing The Cancer Genome Atlas (TCGA) data, we found that the expression of HK3 was closely related to the main clinical features as well as to molecular characteristics. We also predicted that cases with low expression of HK3 were usually malignant entities and were shown to be obvious genomic aberrations of driver oncogenes. At the same time, gene ontology analysis based on significantly related genes in HK3 expression showed that HK3 expression was linked to inflammatory activity and immune response. Additionally, HK3 showed a remarkable trend in predicting the efficacy of immunotherapy for patients receiving Keytruda (PD‐1 monoclonal antibody) treatment.

**Conclusions:**

This is the first comprehensive study to characterize HK3 expression in NSCLC from molecular and clinical aspects.

**Abbreviations**
AUC: area under the curve
GO: gene ontology
NK cell: natural killer cell
ROC: receiver operating characteristic
TCGA: The Cancer Genome Atlas

## 1 BACKGROUND

Non‐small cell lung cancer (NSCLC) is the most common cause for cancer‐induced deaths all over the world.^1^ At present, some progress has been made on comprehensive treatments for NSCLC, including adjuvant radiotherapy, surgical resection, and other treatments. However, patients with NSCLC have bad prognoses because of tumor aggression, relapse, and opposing treatment. Immune‐related mechanisms have great effects on the treatment of NSCLC. What is more, immunotherapeutic strategies are regarded as hopeful directions for treating NSCLC.^2^, ^3^ The most effective immunotherapies are immune checkpoint blockades, especially PD‐1 (programmed death 1) blockade and PD‐L1 (programmed death‐ligand 1) blockade.^4^ Specifically, the PD‐L1 on tumor cells binds to a PD‐1 receptor to initiate the programmed death of T cells by inhibiting their functions and causing immunosuppression.^5^ However, most NSCLCs are refractory to current immunotherapies. This has raised our interest in finding the underlying mechanism of how the immune system regulates the microenvironment of NSCLC to improve immunotherapy benefits.

Another important feature of cancer is metabolic reprogramming, which is characterized by the upregulation of glycolysis; it provides energy and metabolites for cancer cells, which are vital for large‐scale biosynthesis, active cell division, and metastasis.^6^, ^7^, ^8^ Elevated glycolysis is considered an important part of the pernicious phenotype and also a marker of infiltrating cancer. Several studies have paid great attention to exploring the relationship between immune escape and the disorder of energy metabolism during the last decade.^9^, ^10^ Tumor glycolysis, that is, anaerobic glycolysis, affects tumor microenvironment and becomes the main obstacle to successfully targeting cancer with antitumor immune cells and other therapies.^11^ Dysregulating tumor glycolysis could render tumor cells sensitive to natural killer cell (NK cell)‐mediated immunotherapy by upregulating stress‐inducible NKG2DLs (MIC‐A/B) and affecting the acidity of tumor microenvironment (TME), which increases the penetrability or infiltration of the antitumor immune system.^12^ Reportedly, high serum lactate dehydrogenase levels before and during treatment suggest that prognosis is not good.^13^, ^14^

The initial step of glycolysis operates in the presence of hexokinases (HKs).^15^ HK1, HK2, HK3, and HK4 (or GCK) are four types of HK isoenzymes, and they are encoded by different genes. There is proof that HK1 and HK2 are overexpressed in many tumors.^16^, ^17^, ^18^ Moreover, deleting HK2 decreases the proliferation of cancer cells and does not have conspicuous side effects in animal models.^19^ Recently, a study established that HK3 played a functional role in acute promyelocytic leukemia and colorectal cancer.^20^, ^21^

In this study, we analyzed the co‐expression relationship between the glycolytic pathway gene and the immune checkpoint gene and found that the expression of HK3 in tumor tissues may be related to immune status. Based on the data from Tumor Immune Estimation Resource (TIMER) and The Cancer Genome Atlas (TCGA), we studied the relationship between the expression of HK3 and the infiltration of NSCLC. The findings revealed the crucial role of HK3 in NSCLC and showed the potential relationship and the mechanism of interaction between HK3 and tumor immunity. We also obtained and analyzed genomic maps, including somatic mutations and DNA copy numbers. In addition, the result showed that HK3 had an apparent trend in predicting the efficacy of immunotherapy in patients receiving the PD‐1 monoclonal antibody treatment. This is the first comprehensive study to characterize HK3 expression in NSCLC from molecular and clinical aspects.

## 2 MATERIALS AND METHODS

### 2.1 Data collection and patient selection

The data used for this study were mainly from three sources. First, a series of data, including the transcriptome expression data of 566 cases of lung adenocarcinoma (LUAD) and 484 cases of squamous cell carcinoma (LUSC), cases with mutated gene data corresponding to cases with RNA sequence data,^22^ and proteomic analysis based on Reverse Phase Protein Array (RPPA) were downloaded from TCGA. The level 3 data can be obtained from the TCGA directly and used in analyses afterward. The second data source was the cBioPortal for Cancer Genomics, where fraction of the copy number–altered genome (FGA) data was acquired.^23^, ^24^ The third data source was the Wuhan Union Hospital, where human lung cancer tissue specimens were acquired. All specimens used in this study were anonymous. All protocols applying human samples were carried out under the examination and approval of the Ethical Committee of the Huazhong University of Science and Technology. Written informed consent was prepared and delivered by all patients.

### 2.2 Bioinformatic analyses

The R‐3.4.3, SPSS 22, and GraphPad Prism 7 software tools were used to analyze statistics and generate figures. Patient samples from TCGA data sets were analyzed. Gene and immune cell correlations with HK3 expression were studied using Pearson's correlation coefficient (*r*); an absolute *r*‐value of more than 0.3 indicated that the correlation with HK3 was significant. Gene annotation and pathway were investigated using DAVID 6.8.^25^ The biological functions of HK3 were investigated using Gene Set Enrichment Analysis. Gene Set Variation Analysis (GSVA) was deployed to test and verify the relevance of the relationship between HK3 and candidate functions, and it was also used to analyze the relevance of the relationship between HK3 and each immune cell type to determine the effect of HK3 expression on immune cell subsets further. A threshold of an absolute *r*‐value >0.45 and a *P*‐value <.05 were used to select immune cells with obvious correlations with HK3.

### 2.3 IHC and H&E staining

Immunohistochemisty (IHC) and hematoxylin‐eosin (H&E) staining were performed as described previously,^7^ utilizing the following primary antibodies: anti‐CD8A (1:100, Abclonal) and anti‐CD274 (1:200, Abclonal).

### 2.4 Western blotting

Western blotting was performed as described previously^11^ utilizing the following major antibodies: anti‐HK3 (1:500, Abclonal), anti‐CD274 (1:200, Abclonal) and anti‐GAPDH (1:5000, Abclonal).

### 2.5 Reverse transcription‐polymerase chain reaction (RT‐PCR)

The total RNA was extracted by the Trizol Reagent (Invitrogen). SYBR® Premix Ex TaqTM II (Takara Bio, Shiga, Japan) was used to reverse transcribe total RNA into cDNA under the instruction guide of the manufacturer. SYBR Green Mastermix (Takara Bio, Japan) with a Step One Plus Real‐Time PCR system (Applied Biosystems) was used for amplification. The following primer sequences were used: forward: GTGGCAGTGCTGGACGAAGAC; reverse: AGGGTATGGTCGAAGGTGGTCAG.

### 2.6 Statistical analysis

Spearman's correlation analysis was utilized to explore the relationship between continuous variables. The differences of variables between groups were evaluated using the Student *t*‐test and one‐way ANOVA or Pearson's chi‐squared test. The univariate and multivariate Cox proportional hazard models were used to explore the prognostic value of HK3. In addition, the area under the curve (AUC) was ascertained, and the optimal cut‐off value was derived after the creation of the receiver operating characteristic (ROC) curves. Finally, all statistical data were collected and analyzed with the SPSS 24.0 statistical software and *R* project (version 3.4.1). *P* vale < .05 indicated that the difference was significant. Each statistical test was double‐sided.

## 3 RESULTS

### 3.1 The coexpression relationship between the immune checkpoint gene and glycolytic pathway gene

**FIGURE 1:**
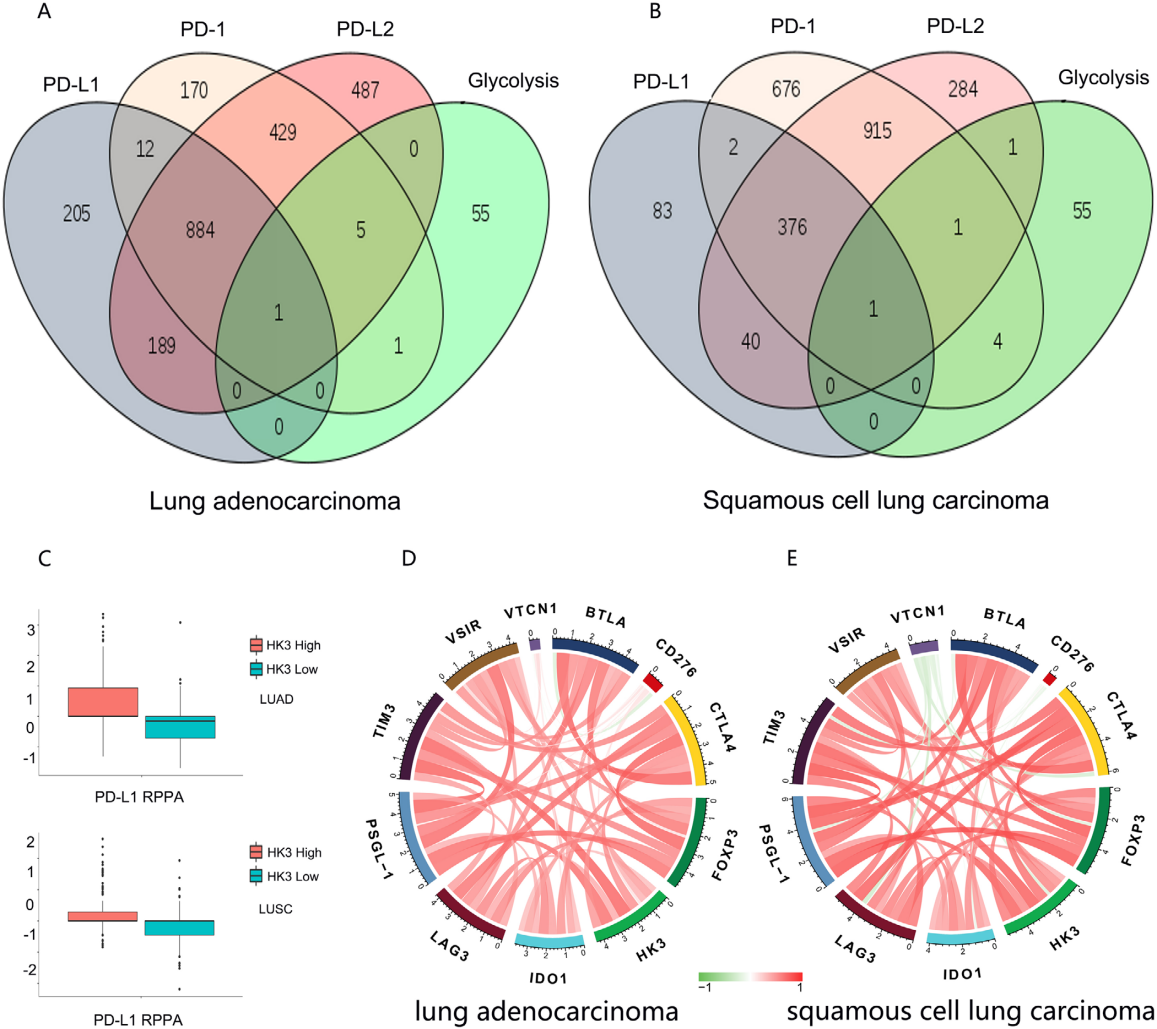
The correlation between immune checkpoint members and HK3 in LUAD and LUSC. A and B, Venn diagram of the number of genes associated with PD‐L1, PD‐L2 PD‐1, and the glycolytic pathway gene in LUAD (A) and LUSC (B) mined from the TCGA database. (C) HK3 expression correlated positively with the PD‐L1 protein level from RPPA data. D and E, The simultaneous cooperation and operation between HK3 and other immune checkpoints in response to tumor‐induced immune in LUAD (D) and LUSC (E)

Previous studies have shown that the glycolytic pathway is involved in tumor immune escape. However, the specific mechanism of how this happens is not clear. Using the TCGA database, we screened for genes that correlated positively with PD‐L1, PD‐1, and PD‐L2 expression (cor > 0.3). We crossed the three sets of genes and obtained genes that correlated positively with the three checkpoints. Then, we crossed the genes of these genes with the glycolytic pathway in kyoto encyclopedia of genes and genomes (KEGG) and found that the first rate‐limiting enzyme, HK3, in the glycolytic pathway correlated positively with the three checkpoints. We performed the same procedure both in LUAD and LUSC and found that HK3 had the same trend in both cancers (Figure 1A,B).

Next, we sought to investigate the relevance of the relationship between the expression of HK3 and PD‐L1 in PD‐L1 RPPA analysis according to the data obtained from the TCGA database. The results of this study indicated that the PD‐L1 protein level was upregulated in the HK3 high expression group (Figure 1C). Previous studies have demonstrated that clinical benefits can be raised through combination therapy by impeding immune checkpoints.^26^, ^27^ Drug treatments aimed at immune checkpoints are still to be assessed in preclinical and clinical trials. In order to deeply study the correlation between HK3 expression and immune checkpoint, the extra genes of immune checkpoints, including CTLA‐4, FOXP3, LAG‐3, and TIM‐3, were also studied in this research. As shown in Figure 1D,E, HK3 in both LUAD and LUSC correlated positively with multiple immune checkpoints.

### 3.2 HK3 expression correlates with immune infiltration levels in NSCLC

**FIGURE 2:**
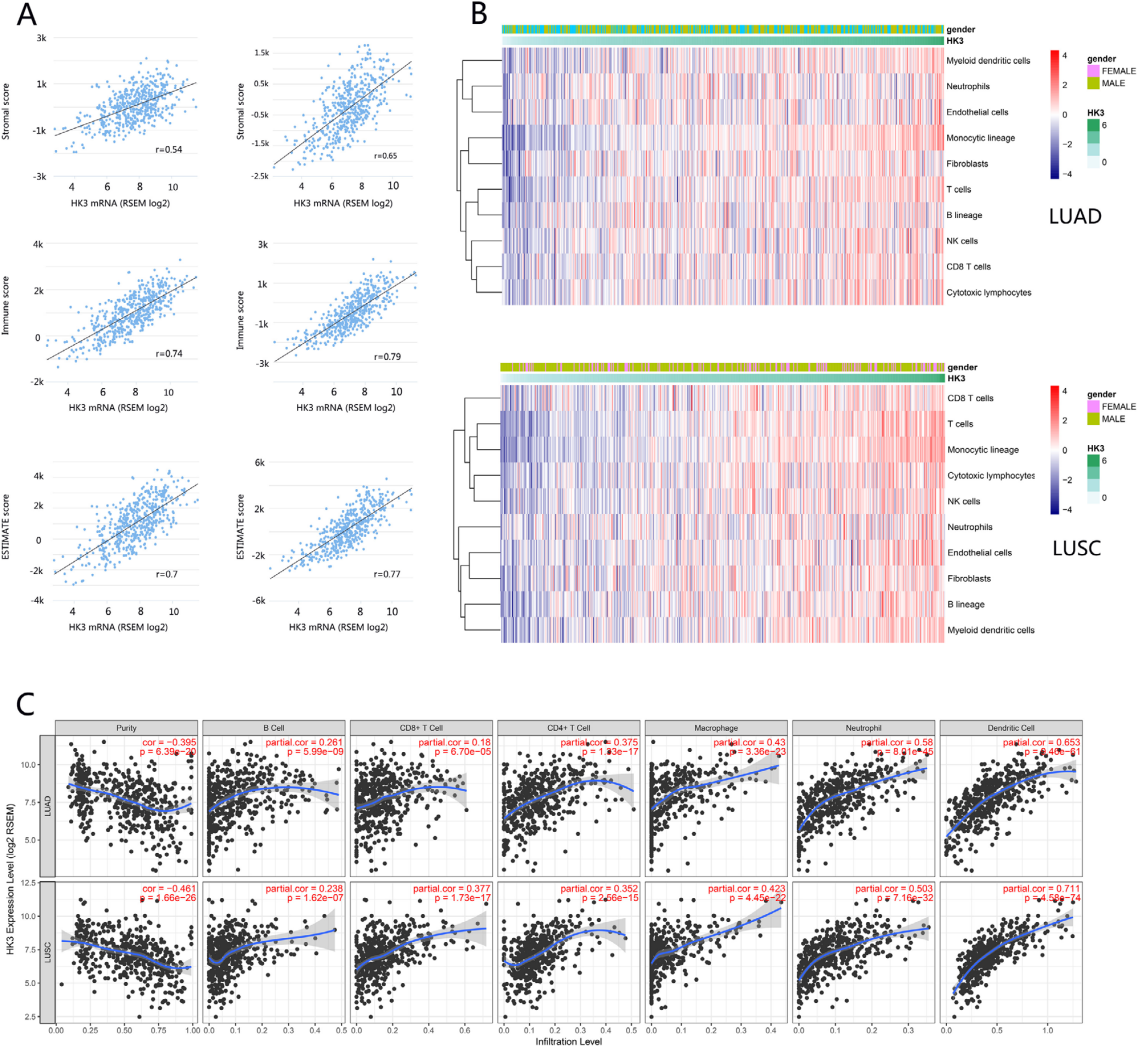
The correlation between HK3 expression and immune infiltration levels in NSCLC. A, Pearson's correlation coefficients between HK3 mRNA expression from the TCGA LUAD and LUSC datasets and ESTIMATE score (*r* = 0.7 in LUAD, *r* = 0.77 in LUSC). B, The relationship between PD‐1 expression and immune cell populations in LUAD and LUSC from the TCGA database. C, The negative correlation between HK3 expression and the purity of tumors and the positive correlation of HK3 expression with infiltrating levels of B cells, CD8+ T cells, CD4+ T cells, macrophages, neutrophils, and dendritic cells in both LUAD and LUSC

Immune cell infiltration of tumor tissue can become independent predictors of the sentinel node status and survival of cancer. Findings from previous clinical trials suggest that the curative effect of the PD‐1 monoclonal antibody is related to the immune infiltration status of tumor tissues.^28^, ^29^ Consequently, we tried to explore the relationship between the expression of HK3 and the level of immune infiltration in NSCLC. The stromal score, immune score, and ESTIMATE score were determined with ESTIMATE, which calculated the immune score for each case by using the data from the TCGA database. As per the results, there were positive and apparent correlations between HK3 expression and stromal score, immune score, and ESTIMATE score in both LUAD and LUSC (Figure 2A), demonstrating that the degree of immune cell infiltration in tumor tissues with high expression of HK3 is also higher.

Next, we focused on the association between HK3 expression and immune cell populations. By using the microenvironment cell populations‐counter method, which has been utilized by some scholars, including Becht et al, the relationship between PD‐1 and immune cell population was explored by analyzing transcriptomic data.^30^ The results showed that there were close relationships between HK3 expression and T cells, myeloid dendritic cells, NK cells, and monocytic lineage (Figure 2B).

To verify the trend we established, we analyzed our data with TIMER. The results revealed that HK3 expression had a negative and observable correlation with the purity of tumors in both LUAD and LUSC (Figure 2C). In addition, HK3 expression correlated significantly negatively with tumor purity in multiple cancer types (Figure S2).

### 3.3 Gene ontology analysis of HK3 in NSCLC

**FIGURE 3:**
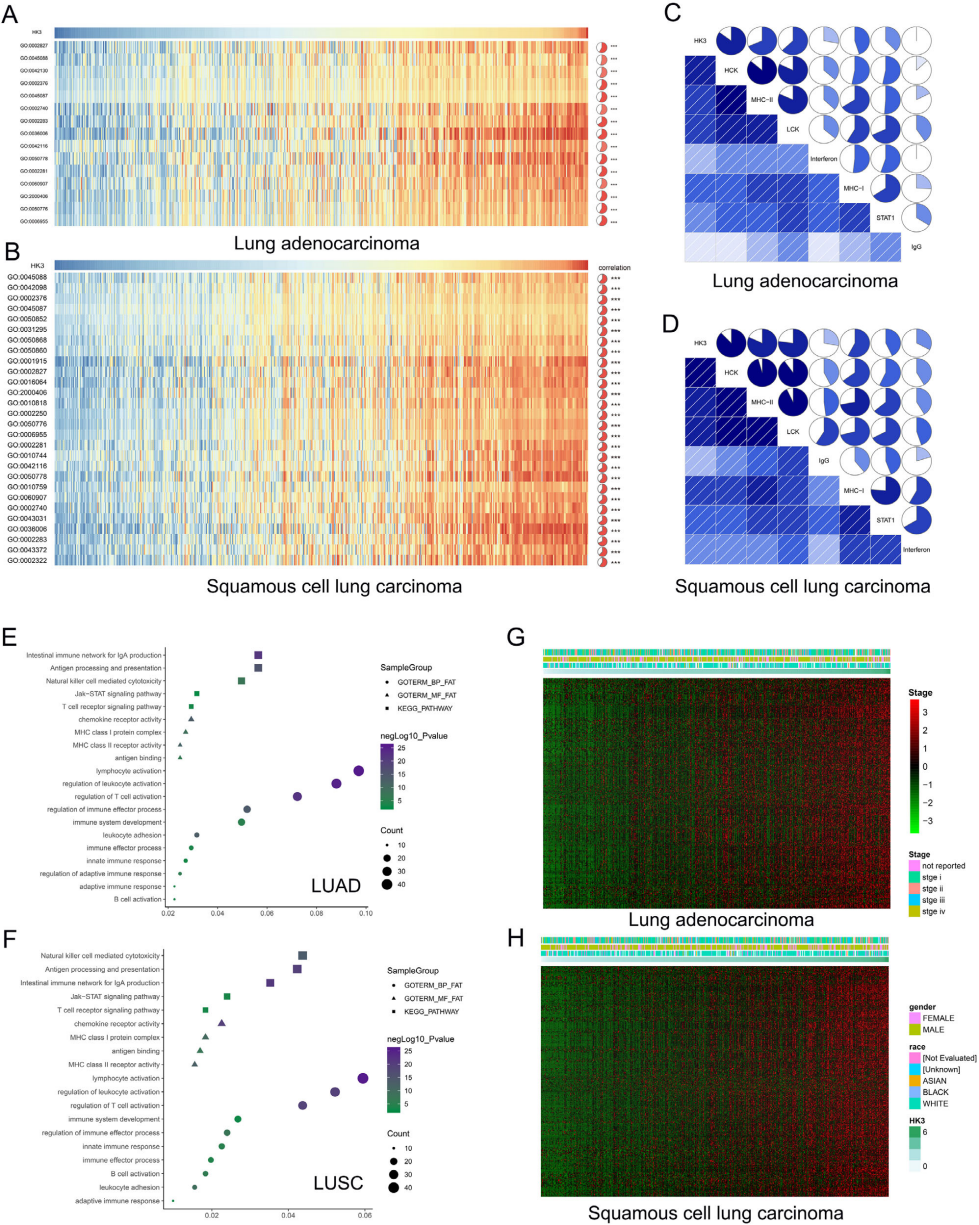
Gene ontology analysis of HK3 in NSCLC. A and B, Gene ontology analysis of the positive correlation bioprocess. Results show that HK3 participated mainly in the reaction of inflammatory responses and immune in LUAD and LUSC. C and D, The positive correlation between most immune‐related genes and the expression of HK3 in LUAD and LUSC according to heatmaps (details regarding related gene information has been provided in the Supporting Information materials). E and F, The correlation between HK3 and T cell immunity in LUAD and LUSC datasets. G and H, The association between HK3 and inflammatory activities in LUAD and LUSC

According to previous results, HK3 potentially plays an essential role in biological functions in NSCLC. To verify this hypothesis, we selected 1467 LUAD genes and 2160 LUSC genes that correlated strongly with HK3, as per Pearson's correlation analysis (Pearson *R* > 0.3), from the TCGA database to conduct analyses afterward. The biological functions of related genes were studied and discussed using gene ontology (GO) analysis in the DAVID Bioinformatics Resources 6.8. Results showed that the genes that correlated positively with HK3 expression in LUAD and LUSC participated in inflammatory and immune responses when the functions of genes were arranged in increasing *P*‐value (Figure 3A,B). To expound the function of HK3 in the immune response in NSCLC, the gene sets associated with immune responses from the AmiGo 2 Web portal were determined, as noted above, and 329 LUAD genes and 407 LUSC genes that had close relationships with HK3 (Pearson *R* > 0.3) were selected and used to create heat maps (Figure 3C,D). Genes with negative correlations were too few, and the data are not displayed.^31^ Hence, HK3 in NSCLC correlated positively with most relevant immune responses.

As the rate‐limiting enzyme for glycolysis, HK3 is dysregulated in various tumors and participates in tumor progression. However, it remains unclear whether HK3 plays a role in the inflammatory activities in NSCLC. To evaluate the relationship between HK3 and the related inflammatory activities of multiple immune cells in NSCLC, we performed GSVA. As per our findings, HK3 correlated positively with these inflammatory activities in both LUAD and LUSC. Significantly, related signals shared by LUAD and LUSC were used to draw heat maps (Figure 3E,F).

For further analysis of the function of HK3 in anti‐tumor immune response, seven associated immune metagenes were evaluated.^32^ According to the result, HK3 expression correlated positively with all seven metagenes (Figure 3G,H). These results also revealed that HK3 was upregulated in the activation of signaling transduction of T cells, macrophages, B lymphocyte‐related metagenes, and antigen‐presenting cells. At the same time, we demonstrated that HK3 played a crucial immune and inflammatory role in LUAD and LUSC.

### 3.4 HK3 expression is associated with clinical and molecular characteristics in NSCLC

**FIGURE 4:**
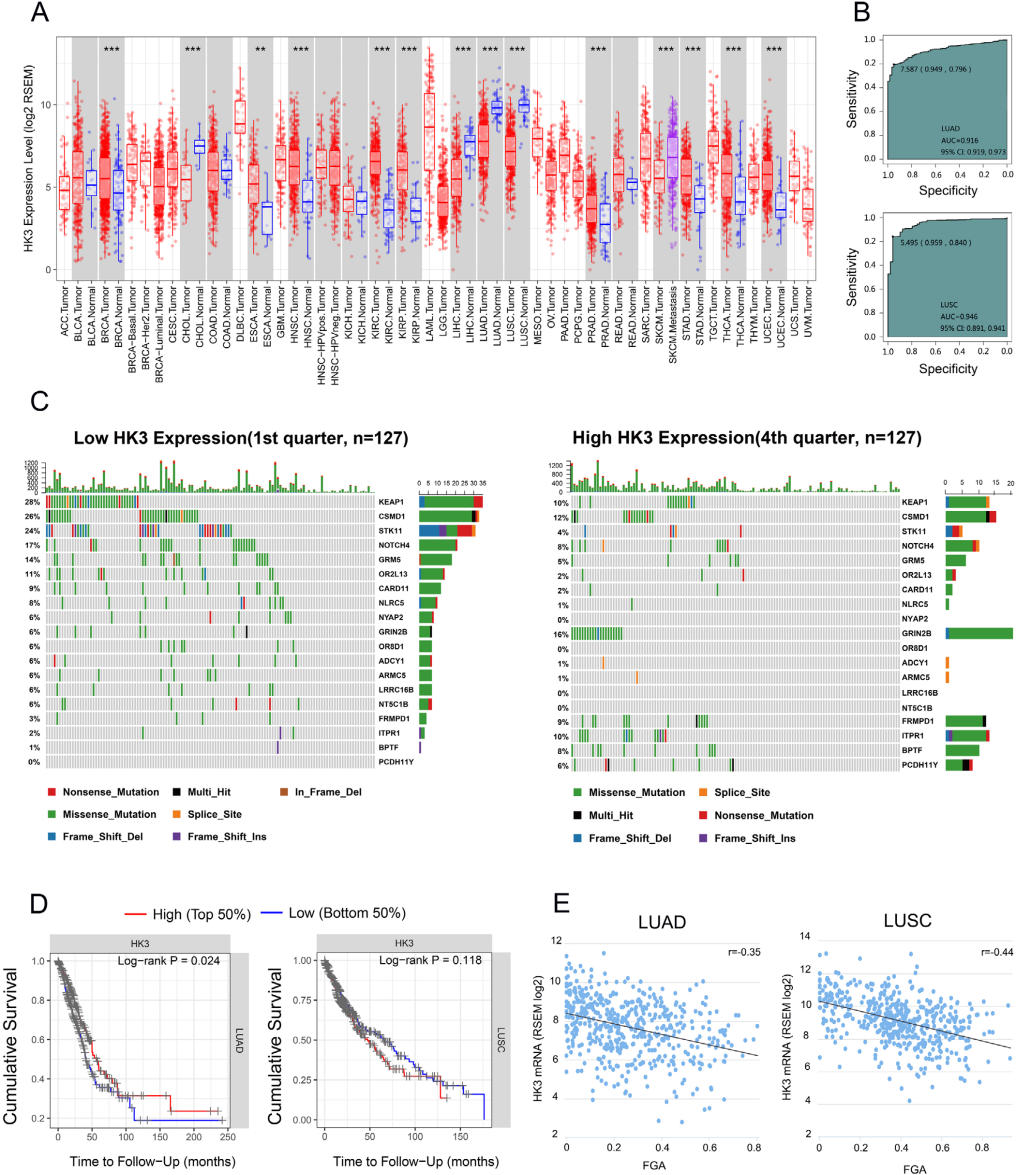
The relationship between HK3 expression and clinical and molecular characteristics in NSCLC. A, Analytical proof of the differences in the levels of human HK3 expression in various types of tumors from the TCGA database using TIMER (*P < .05, **P < .01, ***P < .001). B, ROC curves’ prediction of CMTM6 as a biomarker of LUAD and LUSC. C, The discrepancy in the mutations of somatic cells in LUAD with low and high HK3 expression. D, Comparison of cumulative survival between the high and low expression of HK3 in LUAD and LUSC using TIMER. E, Pearson's correlation coefficients between HK3 mRNA expression from the TCGA LUAD and LUSC datasets and FGA (*r* = −0.35 in LUAD, *r* = −0.44 in LUSC)

For further evaluation of HK3 expression in human cancers, HK3 expression was assessed using RNA‐seq data from multiple malignancies in the TCGA database. TIMER database analysis showed that HK3 expression was considerably higher in both LUAD and LUSC than in normal lung tissues (Figure 4A). Additionally, HK2 was significantly upregulated in LUSC (Figure S1). What is more, the ROC curve was utilized to assess its ability to resolve and identify the HK3 in NSCLC from that in normal tissues. Unexpectedly, the AUC of HK3 expression in the LUAD cohort was 91.6%, and that of HK3 expression in the LUSC cohort was 94.6% (Figure 4B).

Since HK3 associated positively with multiple immune checkpoints, we next investigated the prognostic value of HK3. According to Figure 4D, LUSC patients who had high HK3 expression tumors survived longer, possibly due to the more obvious immune infiltration in the tumor tissues of patients with high HK3 expression.

For the purpose of investigating molecular mechanisms in NSCLC, we analyzed copy number alterations and somatic mutations using data from the cBioPortal datasets. According to the increasing sequence of HK3 expression, samples were divided into a different number of groups, such as two groups or three groups. Parallel analyses were carried out in each type of group, so as to improve the dependability of the findings during the study. After comparing and analyzing the mutation frequency of specimens with low HK3 expression and that of specimens with high HK3 expression, we found that more mutations occurred in STK11, KEAP1, and NOTCH4 in the specimens with low HK3 expression (Figure 4C).

At that point, the changes in somatic copy number were explored in specimens with low and high HK3 expression. Through observation, we found that the correlation between HK3 expression and the FGA (fraction of copy number–altered genome) was negative, which was associated with the genome scope of a copy number profile (*r* = −0.35, *P* < .05 in LUAD and *r* = −0.44, *P* < .05 in LUSC) (Figure 4E).

### 3.5 HK3 plays an effective role in predicting the efficacy of the PD‐1 monoclonal antibody

**FIGURE 5:**
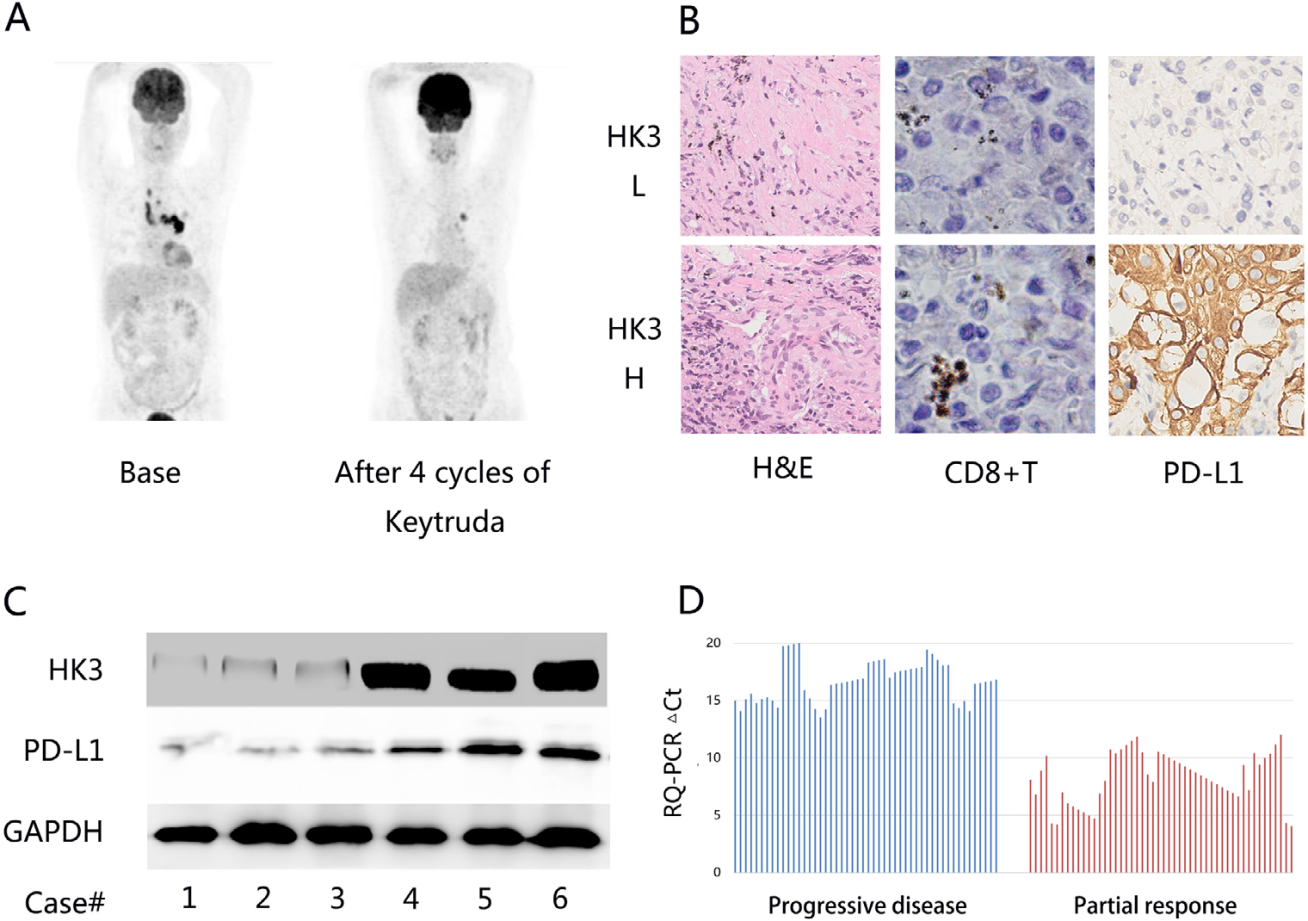
The effective role of HK3 in predicting the efficacy of the PD‐1 monoclonal antibody. A, PET/CT scan detection of a distinct imaging change of a patient treated with Keytruda after four cycles. B, Elevated PD‐L1 and CD8+ T cells in lung cancer tissues. H&E staining was utilized to assess the pathology of patients with lung cancer. C, Western blot analysis of the expression levels of HK3 and PD‐L1 in lung cancer tissues. The relative protein levels were tested through densitometry, and the results showed that all proteins became normal and reached the levels of GAPDH. D, HK3 expression values of tumor tissue specimens from 50 patients with PR and 50 patients with PD before immunotherapy, as determined by reverse transcription‐polymerase chain reaction (RQ‐PCR)

In the 2019 version of the NCCN guidelines, PD‐L1 >50% was used as a cut‐off value for immunotherapy. Additionally, clinical experiments have found that tumor mutation burden, immune cell infiltration, FGA, and STK11 mutations are all factors that influence the prognosis of immunotherapy. To test whether CD8+T cell‐infiltrated HK3 genes can be used as biomarkers to predict immunotherapy efficacy, a retrospective and observational, open study was conducted (ChiCTR1900022601). Under the guidance of CT, 100 cases of patients treated with Keytruda were biopsied by experienced surgical pathologists from the Wuhan Union Medical College Hospital. Detailed clinical characteristics are summarized in Table 1. H&E staining was used to determine whether patients with lung cancer had lymph node metastasis or not. Initially, we focused on one patient who had a significant objective response with a high expression of HK3 after four cycles of treatment with Keytruda (Figure 5A).

**Table 1:** Patients characteristics

	PD	PR
No. of patients	50	50
Median age, years(range)	61.5 (34‐87)	56.5 (24‐85)
Sex		
Male	21	24
Female	29	26
Histology		
Adenocarcinoma	6	5
Squamous	44	45
Line of therapy		
First	50	50
Treatment		
Keytruda	50	50
Stage		
IV	50	50
PS Score		
0	10	7
1	29	34
2	11	9
Metastasis		
Bone	32	40
Brain	8	12
Other	18	12
RQ‐PCR		
Ave. *F* Ct	16.7	8.3
SD	1.77	2.23
Fold Induction	1	333.13

Abbreviations: PD, progression disease; PR, partial response.

For the purpose of further confirming these preliminary findings, we explored the expression of PD‐L1 and CD8+ T cells in patients using immunostaining relative to HK3 expression. Consistent with the bioinformatic results, the immune infiltration components of the low HK3 expression group were different from those of the high HK3 expression group (Figure 5B). In lung cancer patients’ tissues, it could be detected distinctly that when HK3 was losing, the PD‐L1 levels were increased (Figure 5C).

Based on these results, the correlation between HK3 expression and the efficacy of immunotherapy was tested. We received tumor tissue specimens from 50 patients with partial response (PR) and tumor tissue specimens from 50 patients with progressive disease (PD) before immunotherapy to test for HK3 expression values using RQ‐PCR (Figure 5D). Surprisingly, compared with the PD group, there was a 333.13‐fold change in the average HK3 expression value in patients in the PR group. This result strongly suggests that HK3 could be an effective marker for predicting the efficacy of the PD‐1 monoclonal antibody.

## 4 DISCUSSION

With the understanding and acquaintance of tumor immunobiology deepening among researchers, the breakthroughs in the field of tumor immunotherapy in recent years have provided new approaches for cancer therapy.^33^ According to the measurement of IHC, PD‐L1 expression on the surface of tumor cells could act as a predictive factor for distinguishing between patients benefiting from PD‐1 blockade and those not benefiting. However, only some PD‐L1‐positive patients can get a good response. It is, therefore, crucial to find a new biomarker that predicts the response rate of immunotherapy better.

Here, we reveal that HK3 correlated positively with multiple immune checkpoint expressions of mRNA and protein levels in LUAD and LUSC. Through the GO analysis of the biological role of HK3, we found that HK3 performed a crucial role in inflammatory activities and immunological reactions in NSCLC, which was more evident in LUSC. Next, we tried to explore the changes in different genes in a sequence of increasing HK3 expression, observing that FGA events associated negatively with HK3 expression. Finally, we validated our resulting molecular trends in clinical specimens, and immunohistochemistry findings revealed that the composition of CD8+T cells in the low HK3 expression group differed from that in the high HK3 expression group.

**FIGURE 6:**
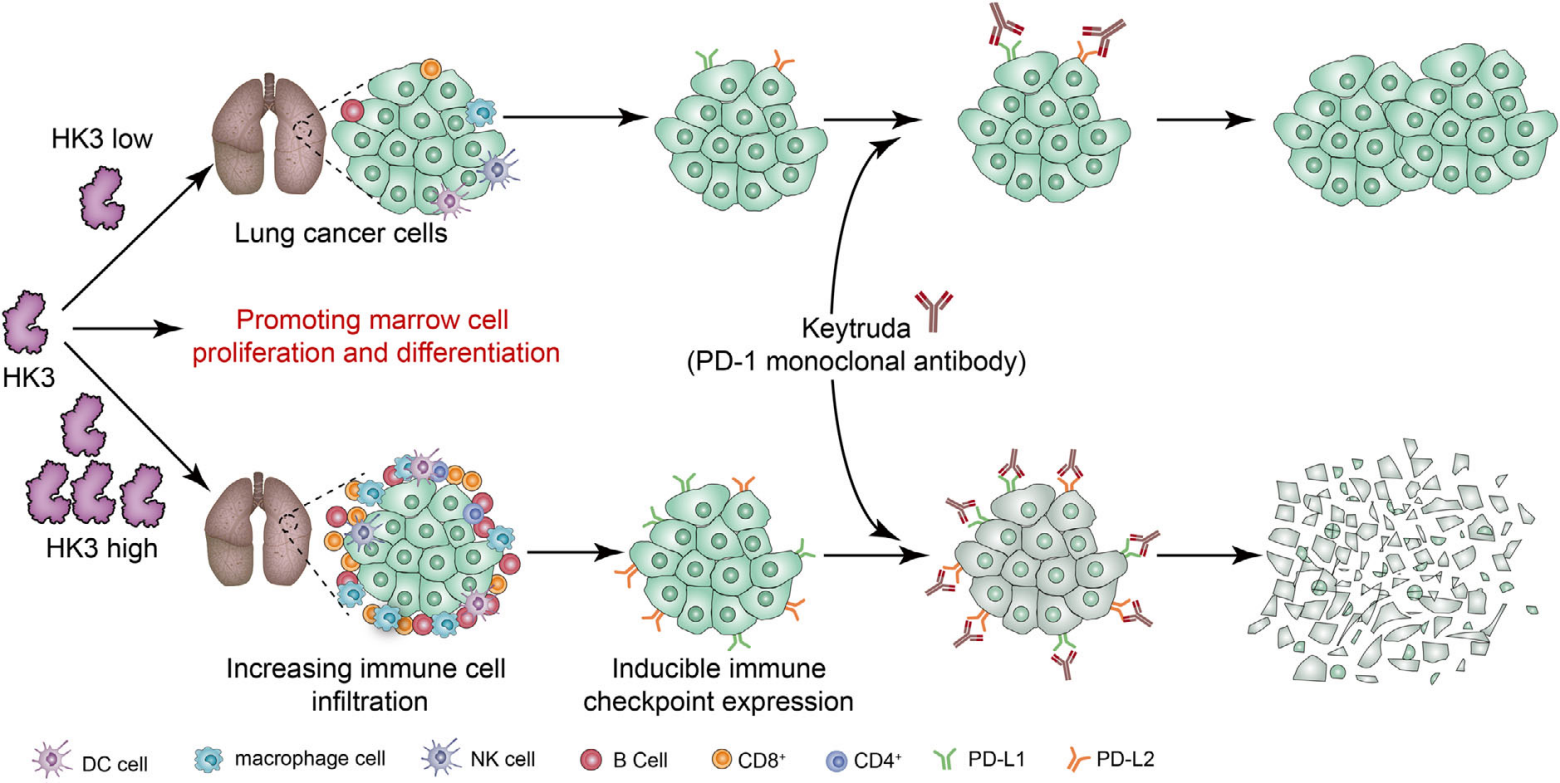
The mechanism suggested by the results of the study

As the first rate‐limiting enzymes of glycolysis, HKs act in various cells, such as tumor cells and immune cells. The analysis of the TCGA database with TIMER showed that HK3 is significantly downregulated in lung cancer tissues, while HK1 and HK2 are significantly upregulated at the same time. So, we speculated that HK3, as an enzyme, does not play a major role in glycolysis in lung cancer cells. There are four isoenzymes in human HK. Type 1 isoenzyme (HK1) is distributed mainly in the brain, HK2 in skeletal muscles, and HK3 in white blood cells. We hypothesized that HK3 is downregulated in tumor cells but not in immune cells, so its expression status is consistent with that of immune cells in NSCLC tissues. The high immune infiltration into tumor tissues prompts tumor cells to defensively upregulate various immune checkpoints to escape the immune system. Therefore, tumor tissues with high expression of HK3 can simultaneously maintain high expression of various immune checkpoints and high immune infiltration. At this time, if various immune checkpoints are suppressed, an excellent immunological antitumor effect can be ascertained (Figure 6).

In this present study, we demonstrated that there was a 333.13‐fold change in the average HK3 expression value in patients in the PR group relative to the PD group. This result strongly suggests that HK3 can be an effective marker for predicting the efficacy of the PD‐1 monoclonal antibody. Our findings indicate that HK3 could serve as an essential biomarker in the immunotherapy of NSCLC.

Some potential predictors have been determined for the response to anti‐PD‐1/PD‐L1 treatments, including tumor‐infiltrating lymphocytes (TILs), mutational landscape and mutational load, and mismatch repair deficiency.^34^ Tumor cells usually express PD‐L1, which could be taken as an adjustment reaction to T‐cell recognition and IFN‐γ stimulation. TMEs have been divided into four types in the past according to PD‐L1 expression and TIL recruitment: (a) PD‐L1(+), with TILs, indicating adaptive immune resistance; (b) PD‐L1(−), without TILs, indicating the occurrence of immune ignorance; (c) PD‐L1(+), without TILs, indicating the emergence of intrinsic induction; (d) PD‐L1(−), with TILs, indicating the functioning of other suppressors on immune tolerance. A type I patient could obtain a better response. Recent investigations indicate that IFN‐γ is an important promoter of the programmed PD‐L1 expression in cancer cells and host cells, and the infiltrating data line of T cells in tumors can raise the possibility of a response to anti‐PD‐1 therapies, including pembrolizumab.^35^ A clinical trial on NSCLC patients who received treatment with immunotherapy revealed that patients who had no durable benefit had the highest copy number–altered genome fragments, but the variants in EGFR and STK11 related to benefit were lacking.^36^ Here, we found that HK3 gene expression correlated positively with a variety of biomarkers for predicting the effectiveness of the PD‐1 monoclonal antibody, including immune checkpoints, immune cell infiltration, FGA, and IFN‐γ signal activity, and has achieved promising predictive results in a large number of immunotherapeutic patient specimens.

To sum up, by analyzing the data of transcriptomes and genomes, we showed that HK3 was not only downregulated in LUAD and LUSC but also cooperated and operated with other immune checkpoints at the same time. In addition, HK3 expression correlated positively with T‐cell activation and antitumor immunity. Furthermore, HK3 is correlated with the outcomes of patients receiving immunotherapy in clinical practice. The findings of this study suggest that HK3 is a potential new biomarker to guide clinical decision‐making for immunotherapy.

## 5 CONCLUSIONS

Our findings show that HK3 is synergistic with multiple immune checkpoints, T‐cell activation, and antitumor immunity. Additionally, HK3 expression correlated positively with the outcomes of patients receiving immunotherapy in clinical practice. The findings of this study make a case for the establishment of HK3 as a potential new biomarker to guide clinical decision‐making for immunotherapy.

## CONFLICT OF INTEREST

The authors declare that there is no conflict of interest that could be perceived as prejudicing the impartiality of the research reported.

## Supporting information

Supporting Information

Supporting Information

## Data Availability

The data utilized in this study are available in the TCGA databases.

## References

[1] Miller KD , Nogueira L , Mariotto AB , et al. Cancer treatment and survivorship statistics, 2019. CA Cancer J Clin. 2019;69(5):363‐385. 31184787 10.3322/caac.21565

[2] Goldberg SB , Gettinger SN , Mahajan A , et al. Pembrolizumab for patients with melanoma or non‐small‐cell lung cancer and untreated brain metastases: early analysis of a non‐randomised, open‐label, phase 2 trial. Lancet Oncol. 2016;17(7):976‐983. 27267608 10.1016/S1470-2045(16)30053-5 PMC5526047

[3] Vansteenkiste JF , Cho BC , Vanakesa T , et al. Efficacy of the MAGE‐A3 cancer immunotherapeutic as adjuvant therapy in patients with resected MAGE‐A3‐positive non‐small‐cell lung cancer (MAGRIT): a randomised, double‐blind, placebo‐controlled, phase 3 trial. Lancet Oncol. 2016;17(6):822‐835. 27132212 10.1016/S1470-2045(16)00099-1

[4] Sznol M , Chen L . Antagonist antibodies to PD‐1 and B7‐H1 (PD‐L1) in the treatment of advanced human cancer–response. Clin Cancer Res. 2013;19(19):5542. 24048329 10.1158/1078-0432.CCR-13-2234 PMC6101650

[5] Dong H , Zhu G , Tamada K , Chen L . B7‐H1, a third member of the B7 family, co‐stimulates T‐cell proliferation and interleukin‐10 secretion. Nat Med. 1999;5(12):1365‐1369. 10581077 10.1038/70932

[6] Pavlova NN , Thompson CB . The emerging hallmarks of cancer metabolism. Cell Metabol. 2016;23(1):27‐47. 10.1016/j.cmet.2015.12.006 PMC4715268 26771115

[7] Liu J , Gao L , Zhang H , et al. Succinate dehydrogenase 5 (SDH5) regulates glycogen synthase kinase beta‐beta‐catenin‐mediated lung cancer metastasis. J Biol Chem. 2013;288(41):29965‐29973. 23983127 10.1074/jbc.M113.450106 PMC3795294

[8] Zong Y , Li Q , Zhang F , et al. SDH5 depletion enhances radiosensitivity by regulating p53: a new method for noninvasive prediction of radiotherapy response. Theranostics. 2019;9(22):6380‐6395. 31588224 10.7150/thno.34443 PMC6771232

[9] Husain Z , Seth P , Sukhatme VP . Tumor‐derived lactate and myeloid‐derived suppressor cells: linking metabolism to cancer immunology. Oncoimmunology 2013;2(11):e26383. 24404426 10.4161/onci.26383 PMC3881600

[10] Ohashi T , Akazawa T , Aoki M , et al. Dichloroacetate improves immune dysfunction caused by tumor‐secreted lactic acid and increases antitumor immunoreactivity.Int J Cancer. 2013;133(5):1107‐1118. 23420584 10.1002/ijc.28114

[11] Tuo Z , Zong Y , Li J , et al. PD‐L1 regulation by SDH5 via beta‐catenin/ZEB1 signaling. Oncoimmunology 2019;8(12):1655361. 31741753 10.1080/2162402X.2019.1655361 PMC6844322

[12] Fu D , Geschwind JF , Karthikeyan S , et al. Metabolic perturbation sensitizes human breast cancer to NK cell‐mediated cytotoxicity by increasing the expression of MHC class I chain‐related A/B. Oncoimmunology 2015;4(3):e991228. 25949910 10.4161/2162402X.2014.991228 PMC4404839

[13] Diem S , Kasenda B , Spain L , et al. Serum lactate dehydrogenase as an early marker for outcome in patients treated with anti‐PD‐1 therapy in metastatic melanoma. Br J Cancer. 2016;114(3):256‐261. 26794281 10.1038/bjc.2015.467 PMC4742588

[14] Martens A , Wistuba‐Hamprecht K , Geukes Foppen M , et al. Baseline peripheral blood biomarkers associated with clinical outcome of advanced melanoma patients treated with ipilimumab. Clin Cancer Res. 2016;22(12):2908‐2918. 26787752 10.1158/1078-0432.CCR-15-2412 PMC5770142

[15] Wilson JE . Isozymes of mammalian hexokinase: structure, subcellular localization and metabolic function. J Exp Biol. 2003;206(Pt 12):2049‐2057. 12756287 10.1242/jeb.00241

[16] Wang L , Xiong H , Wu F , et al. Hexokinase 2‐mediated Warburg effect is required for PTEN‐ and p53‐deficiency‐driven prostate cancer growth. Cell Rep. 2014;8(5):1461‐1474. 25176644 10.1016/j.celrep.2014.07.053 PMC4360961

[17] Palmieri D , Fitzgerald D , Shreeve SM , et al. Analyses of resected human brain metastases of breast cancer reveal the association between up‐regulation of hexokinase 2 and poor prognosis. Mol Cancer Res. 2009;7(9):1438‐1445. 19723875 10.1158/1541-7786.MCR-09-0234 PMC2746883

[18] Smith TA . Mammalian hexokinases and their abnormal expression in cancer. Br J Biomed Sci. 2000;57(2):170‐178. 10912295

[19] Garcia SN , Guedes RC , Marques MM . Unlocking the potential of HK2 in cancer metabolism and therapeutics. Curr Med Chem. 2019;26(41):7285‐7322. 30543165 10.2174/0929867326666181213092652

[20] Federzoni EA , Humbert M , Torbett BE , Behre G , Fey MF , Tschan MP . CEBPA‐dependent HK3 and KLF5 expression in primary AML and during AML differentiation. Sci Rep. 2014;4:4261. 24584857 10.1038/srep04261 PMC3939455

[21] Pudova EA , Kudryavtseva AV , Fedorova MS , et al. HK3 overexpression associated with epithelial‐mesenchymal transition in colorectal cancer. BMC Genomics. 2018;19(suppl 3):113. 29504907 10.1186/s12864-018-4477-4 PMC5836836

[22] Cancer Genome Atlas Research Network . Comprehensive molecular profiling of lung adenocarcinoma. Nature. 2014;511(7511):543‐550. 25079552 10.1038/nature13385 PMC4231481

[23] Gao J , Aksoy BA , Dogrusoz U , et al. Integrative analysis of complex cancer genomics and clinical profiles using the cBioPortal. Sci Signal. 2013;6(269):pl1. 23550210 10.1126/scisignal.2004088 PMC4160307

[24] Cerami E , Gao J , Dogrusoz U , et al. The cBio cancer genomics portal: an open platform for exploring multidimensional cancer genomics data. Cancer Discov. 2012;2(5):401‐404. 22588877 10.1158/2159-8290.CD-12-0095 PMC3956037

[25] Huang da W , Sherman BT , Lempicki RA . Systematic and integrative analysis of large gene lists using DAVID bioinformatics resources. Nat Protoc. 2009;4(1):44‐57. 19131956 10.1038/nprot.2008.211

[26] Boutros C , Tarhini A , Routier E , et al. Safety profiles of anti‐CTLA‐4 and anti‐PD‐1 antibodies alone and in combination. Nat Rev Clin Oncol. 2016;13(8):473‐486. 27141885 10.1038/nrclinonc.2016.58

[27] Nolan E , Savas P , Policheni AN , et al. Combined immune checkpoint blockade as a therapeutic strategy for BRCA1‐mutated breast cancer. Sci Transl Med. 2017;9(393). 10.1126/scitranslmed.aal4922 PMC5822709 28592566

[28] Jacquelot N , Roberti MP , Enot DP , et al. Predictors of responses to immune checkpoint blockade in advanced melanoma. Nat Commun. 2017;8(1):592. 28928380 10.1038/s41467-017-00608-2 PMC5605517

[29] Cottrell TR , Thompson ED , Forde PM , et al. Pathologic features of response to neoadjuvant anti‐PD‐1 in resected non‐small‐cell lung carcinoma: a proposal for quantitative immune‐related pathologic response criteria (irPRC). Ann Oncol. 2018;29(8):1853‐1860. 29982279 10.1093/annonc/mdy218 PMC6096736

[30] Becht E , Giraldo NA , Lacroix L , et al. Estimating the population abundance of tissue‐infiltrating immune and stromal cell populations using gene expression. Genome Biol. 2016;17(1):218. 27765066 10.1186/s13059-016-1070-5 PMC5073889

[31] Guan X , Zhang C , Zhao J , Sun G , Song Q , Jia W CMTM6 overexpression is associated with molecular and clinical characteristics of malignancy and predicts poor prognosis in gliomas. EBioMedicine. 2018;35:233‐243. 30131308 10.1016/j.ebiom.2018.08.012 PMC6156716

[32] Rody A , Holtrich U , Pusztai L , et al. T‐cell metagene predicts a favorable prognosis in estrogen receptor‐negative and HER2‐positive breast cancers. Breast Cancer Res. 2009;11(2):R15. 19272155 10.1186/bcr2234 PMC2688939

[33] Ma W , Gilligan BM , Yuan J , Li T . Current status and perspectives in translational biomarker research for PD‐1/PD‐L1 immune checkpoint blockade therapy. J Hematol Oncol. 2016;9(1):47. 27234522 10.1186/s13045-016-0277-y PMC4884396

[34] Luchini C , Bibeau F , Ligtenberg MJL , et al. ESMO recommendations on microsatellite instability testing for immunotherapy in cancer, and its relationship with PD‐1/PD‐L1 expression and tumour mutational burden: a systematic review‐based approach. Ann Oncol. 2019;30(8):1232‐1243. 31056702 10.1093/annonc/mdz116

[35] Ayers M , Lunceford J , Nebozhyn M , et al. IFN‐gamma‐related mRNA profile predicts clinical response to PD‐1 blockade. J Clin Invest. 2017;127(8):2930‐2940. 28650338 10.1172/JCI91190 PMC5531419

[36] Rizvi H , Sanchez‐Vega F , La K , et al. Molecular determinants of response to anti‐programmed cell death (PD)‐1 and anti‐programmed death‐ligand 1 (PD‐L1) blockade in patients with non‐small‐cell lung cancer profiled with targeted next‐generation sequencing. J Clin Oncol. 2018;36(7):633‐641. 29337640 10.1200/JCO.2017.75.3384 PMC6075848

