# Supplementary material for: HK3 is correlated with immune infiltrates and predicts response to immunotherapy in non‐small cell lung cancer
Source: Clin Transl Med. 2020 May 7;10(1):319–30. doi: 10.1002/ctm2.6 (PMC7240846; doi:10.1002/ctm2.6)
Supplement: Supplementary file 2 — Supporting Information [file CTM2-10-319-s002.docx]

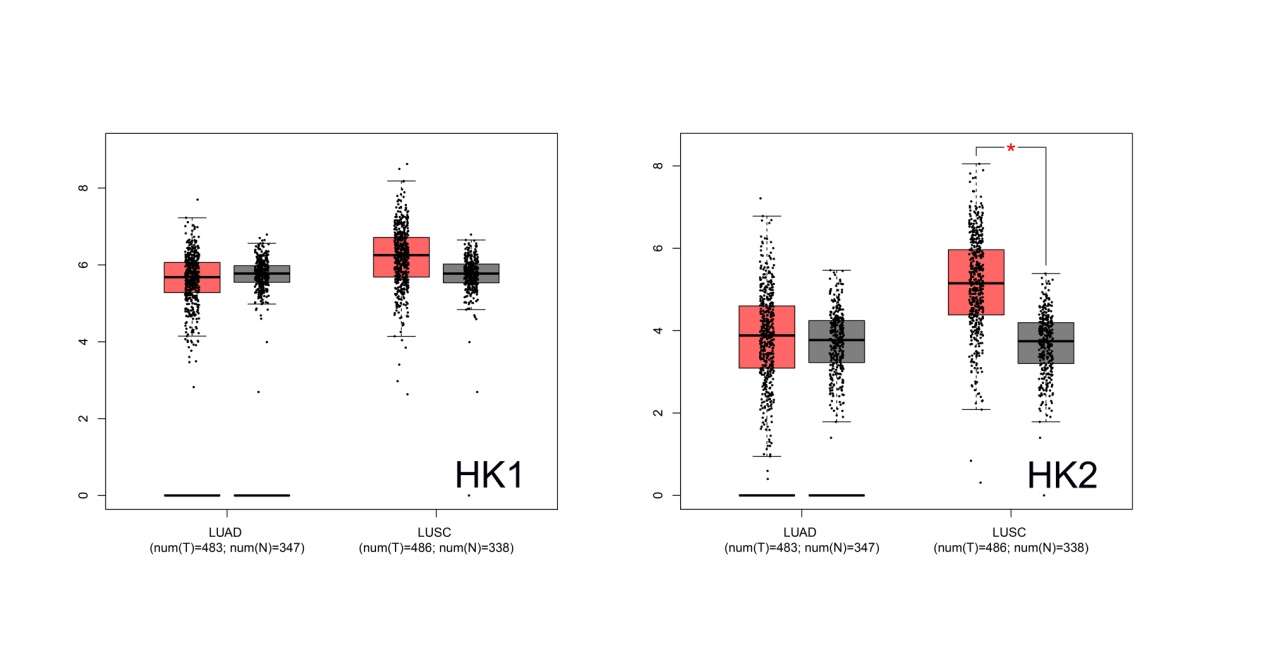


Supplementary Fig. S1 HK2 was significant up-regulation in LUSC.


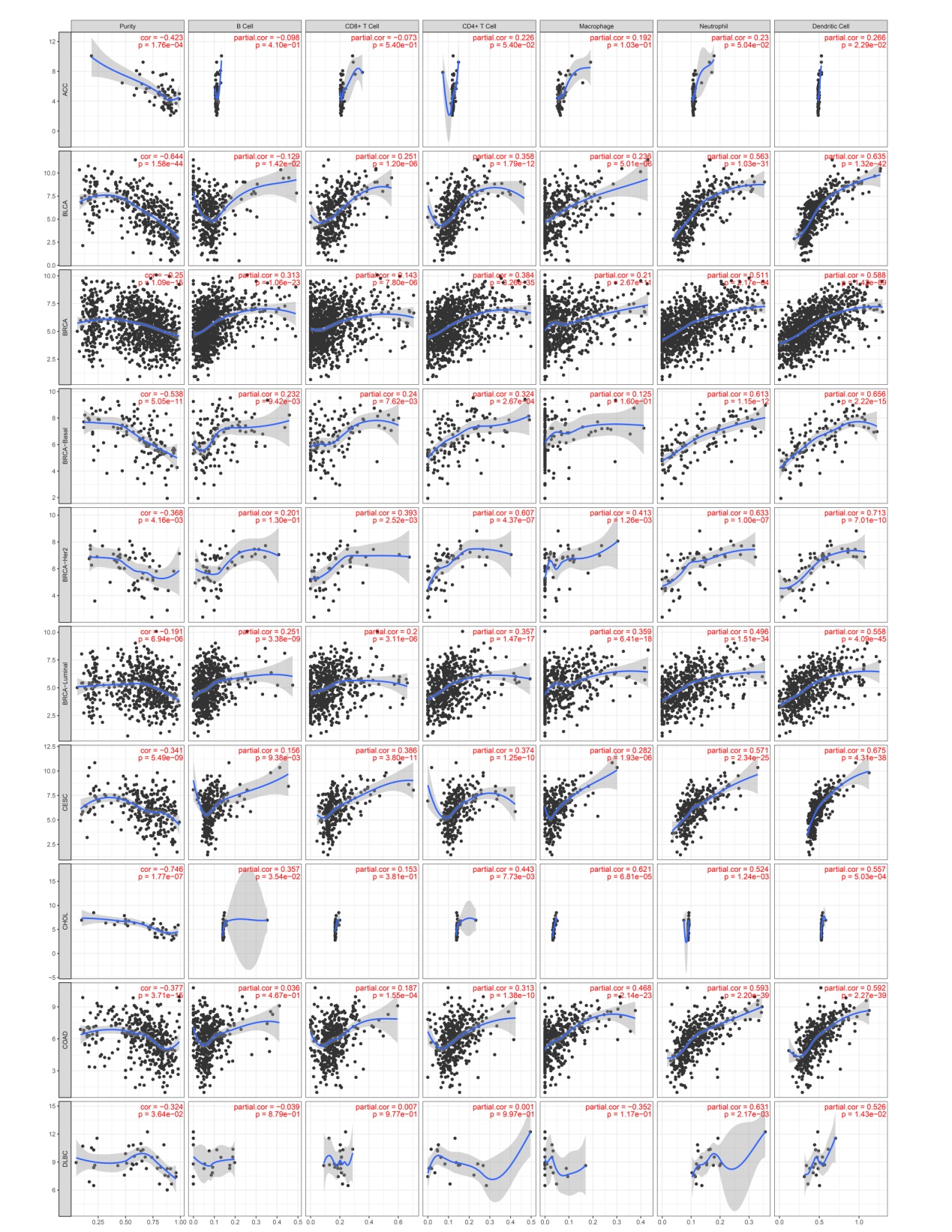


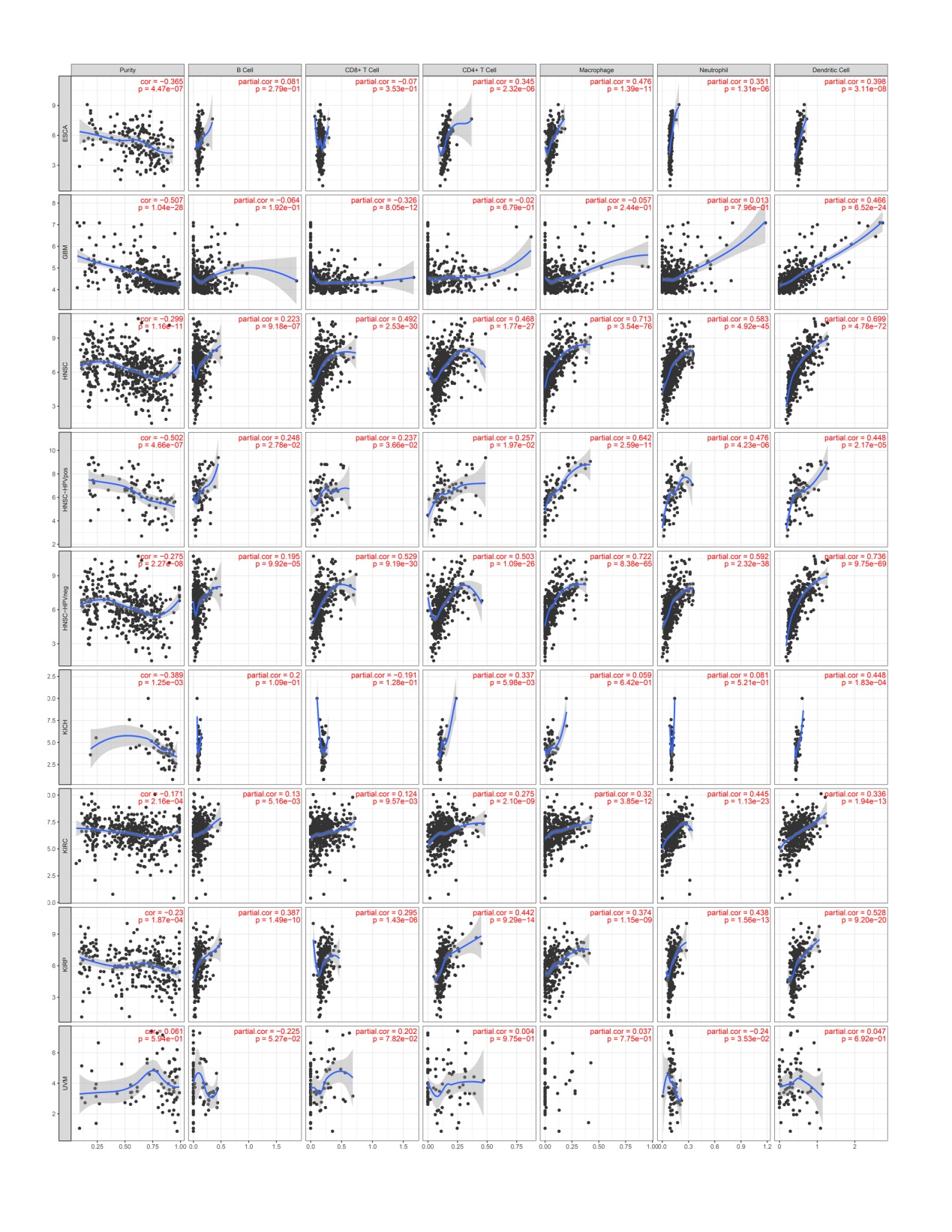


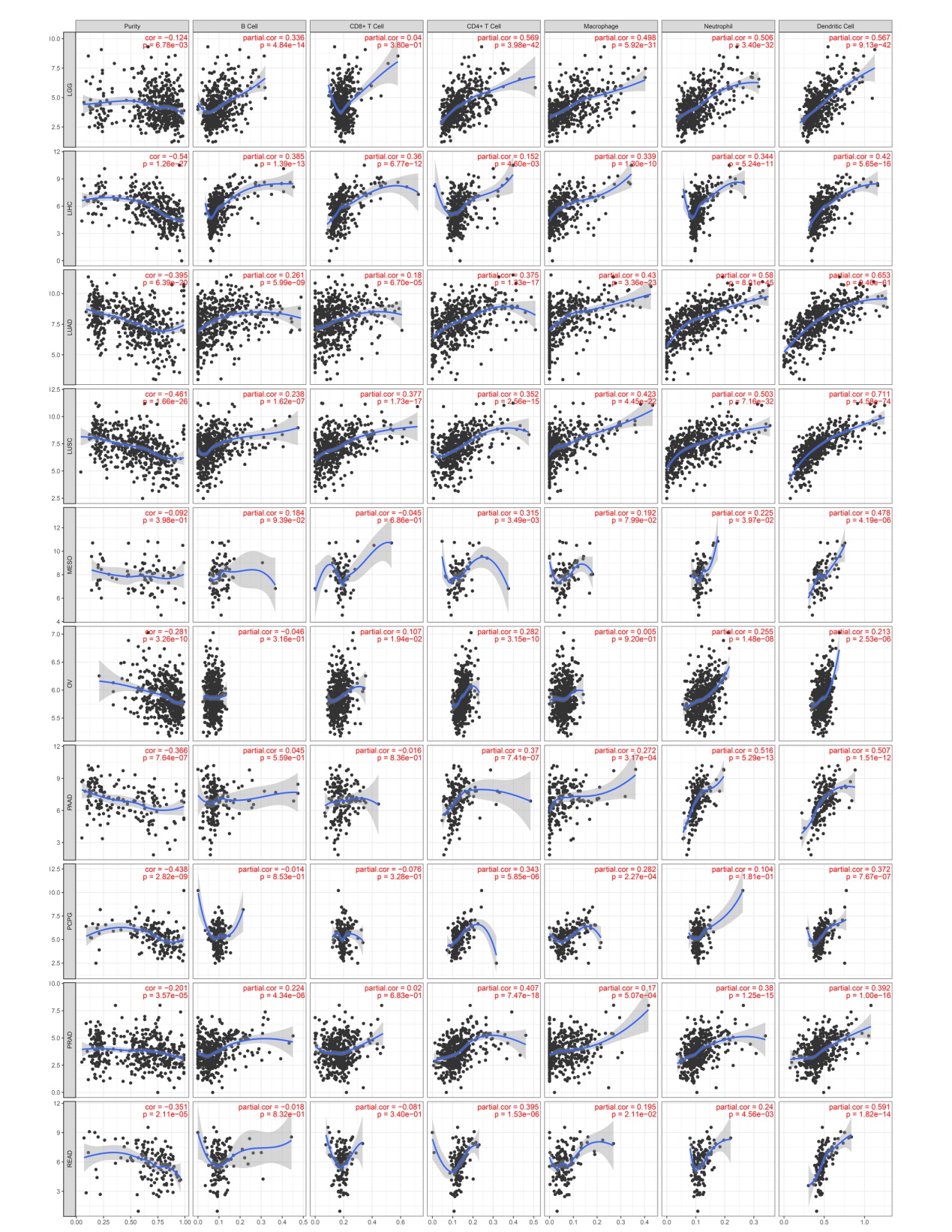


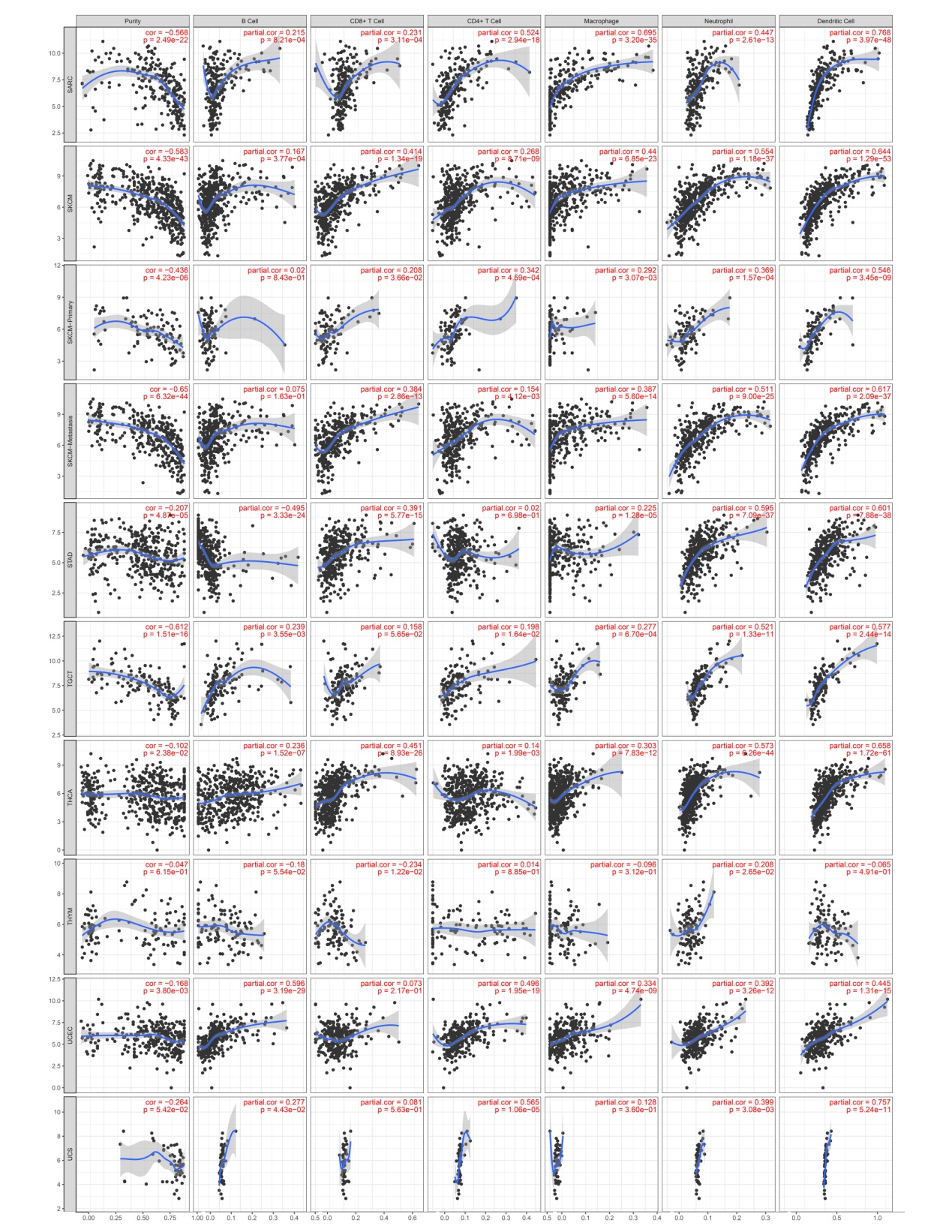


Supplementary Fig. S2 . HK3 expression has significant correlations with tumor purity in mutiply cancer types.
